# Closing out 10 years

**Published:** 2019-11-28

**Authors:** Marcel D’Eon

**Affiliations:** 1 University of Saskatchewan, Saskatchewan, Canada

This issue marks a historical point in the life of the CMEJ: this is the final issue in our 10^th^ year of publication. This issue’s artwork, Head, by O’Neill, used small objects to create a larger, coherent picture similar in style to pointillism. It reminds me of the broad sweep of CMEJ history: understanding many of the events over the first 10 years requires stepping back and seeing the patterns from a distance. From the humble beginnings in 2010, CMEJ has grown from publishing two issues and 90 pages per year to now publishing up to four issues and more than 400 pages per year. The number of days it takes us to give an initial decision went from over 200 to well under 100 even while the number of submissions has increased from 25 per year in the early years to over 150. We now translated all abstracts to make them available in both languages and so more legitimately lay claim to being a Canadian journal. We have moved from a University of Calgary-based publication to a self- publishing academic journal managed by a consortium of five national medical education organizations (The Association of Faculties of Medicine Canada, The Canadian Association for Medical Education, The College of Family Physicians Canada, The Medical Council of Canada, and The Royal College of Physicians and Surgeons). For years our funding was patched together, but now with a three-year grant from the Social Science and Humanities Research Council (Aid to Scholarly Journals) and a solid business plan, we have greater financial stability and respected governance. Each event and accomplishment in our history, however small it may seem, has brought us closer to our goal of being a pre-eminent medical education journal for Canadian scholars and scholarship.

As we continue to grow and expand, new and exciting things are on the horizon for CMEJ. I look forward to the adventures as this journal enters its second decade of scholarly activity in medical education.

In this issue we present a varied array of topics and formats, all interesting studies that can help move medical education forward.

“Does watching a movie improve empathy? A cluster randomized controlled trial” by Ahmadzadeh et al is a study that examined how watching a movie about the patient-physician relationship alone or in combination with a three-hour communication skills training workshop improved the empathy scores of medical students. One hundred and thirty-three medical students participated in one of four groups. The authors used a linear mixed effect model to analyze the effect of intervention across groups considering the effects of other significant variables. All three intervention groups showed an immediate positive effect on empathy scores. However, the improvement effect remained significant only in two of the groups one month later, one of which was the movie and workshop combined.

“Medical Assistance in Dying: the opinions of medical trainees in Newfoundland and Labrador. A cross- sectional study” by McCarthy and Seal explored the opinions of medical trainees in Newfoundland and Labrador regarding MAiD. They distributed a survey to all 570 under- and post-graduate medical trainees at Memorial University. Of the 124 trainees who completed the survey (response rate of 21.8%), 90% supported the legalization of MAiD in Canada. While nearly 60% stated they would serve their patients’ wishes, they also favoured assisted suicide over active euthanasia. Level of training and religious affiliation were associate with support for MAiD.

“Disadvantaged patient populations: a theory- informed education needs assessment in an urban teaching hospital” by Baker and her team used a critical discourse analysis to explore the meanings and effects of disadvantaged patient populations (DPP). They analyzed transcripts from 15 focus groups with trainees, staff, and patients and learned that 1) disadvantaged patients require care above what is normal; 2) the system is to blame for failures in serving disadvantaged patients; and 3) labeling patients is problematic and stigmatizing. Patients both appreciated that the DPP label allowed better access to care, but also felt “othered” at the same time. They suggested theory-informed educational practices to help improve care for DPP.

“Assessing the quality of feedback to general internal medicine residents in a competency-based environment” by Marcotte and her team describe their investigation of the quality of feedback in General Internal Medicine (GIM), by comparing workplace-based assessment (WBA) and In Training Evaluation Reports (ITERs). They predicted that WBAs would improve feedback to support the development of competence. Over a three-year period, they gathered data from focus groups, interviews, and surveys that compared WBA and ITERs. Overall rates of actionable feedback, for both ITERs and WBAs were low (26%), with only 9% of the total providing an improvement strategy. They found that residents and preceptors both believed the narrative component of feedback was more constructive and effective than numerical scores.

“Managing cognitive load in simulations: exploring the role of simulation technologists” written by Sibbald and team used cognitive load theory to explore the impact of technologists on instructors, identifying sources of instructor cognitive load with and without a technologist present. They collected data from 56 simulations for postgraduate emergency medicine residents, 14 without a technologist (42 with one). After each session, the instructor and simulation technologist (if present) import data. Instructors rated the level of their cognitive load identical, regardless of whether technologists were present. Interestingly, instructors experienced reduced cognitive load related to the simulator and technical resources when technologists were present allowing the instructor to focus more on observing the learner(s) and modifying the simulation accordingly.

Sanaee with the University of Toronto in “Medical education reform: a catalyst for strengthening the health system”, presents an important argument for medical educators and health system reformers. Sanaee tries to demonstrate that through a health systems framework, Competence by Design (CBD) provides a medium for health systems reform. Implications of effective implementation of CBD may include staffing shortages in academic hospitals, annual variation in medical education financing, new roles for clinician teachers, and greater demand for human health resource surveillance and patient outcome monitoring and analysis. These can be conceptualized as opportunities to improve coordination, harmonization, and system responsiveness.

“Supporting early academic family medicine careers with the clinician scholar enhanced-skills program” by Lacasse and her team describes the development and evaluation strategy of Laval University’s Clinical Skills Program that trains clinician researchers/educators/leaders for academic family practice. They used Kern’s model for program development and a program-oriented approach for program evaluation. Seven graduates and 14 non- graduates of the program participated in the evaluation. While the evaluation was quite positive, there were suggestions for improvement such as project-based learning with learner-centered objectives, relevant and authentic learning and assessment, and multi-level program evaluation approach.

In “Status of global health fellowship training in the United States and Canada”, Evensen and her international team note the increasing numbers of residency graduates seeking global health (GH) fellowship training but lament the lack of clarity in training options. Using a web-based tool, they surveyed program directors or designates from 85 GH fellowships from which they garnered 50 responses. Most commonly, fellowships were 24 months in duration with a median size of one fellow per year. Funding and lack of qualified applicants were substantial challenges. Most programs were funded through fellow billing for patient care or other means of self-support. The number of U.S. and Canadian GH fellowship programs has nearly doubled between 2010 and 2017 but information about them is not readily available.

“Transitioning to competency-based medical education: impact of educational interventions on internal medicine residents’ understanding of the purpose and process” by Daniels and team from the University of Alberta describe the measures they took to better inform residents of the purpose of and processes used in Competency by Design. They report on the use of short orientation videos and an overview of the learning objectives for each level of training.

“A student affairs podcast as novel communications tool” by Frayha and her team described the use of a podcast on topics relevant to the medical student experience. In just a short time, there have been over 20,000 downloads. Ninety-five percent of survey respondents indicated they would recommend this podcast to others. Given the mission of student affairs offices to advise, mentor, and educate students, this series of podcasts is an exciting innovation for all medical schools to consider.

“A resident-led clinic that promotes the health of refugee women through advocacy and partnership” by Stairs and her team from Dalhousie describes a longitudinal global health experiences to promote cultural competency and a commitment to caring for underserved populations. Obstetrics and gynecology residents have partnered with the Halifax Newcomer Health Clinic to provide education and medical care to refugee women. This resident-led initiative meets the care needs of an underserved population while promoting resident engagement in health advocacy.

Dr. Wilbur ask the question, “Should scholar be the new interprofessional competency?” With the almost universal use of evidence-based practices as a framework, understanding the evidence base for other professions is crucial. Hence, scholarship plays a crucial role in interprofessional shared decision- making.

In “A definition of coaching in medical education” Landreville and co-authors note that coaching differs from teaching and mentoring but lacks a clear operational definition. They suggest that coaching is a process that guides a learner towards performance improvement. This process is important since coaching is central to competence based medical education.

Finally, “Burnout” by Ranpara informs and entertains us with the stressed and rewarding life of the resident. Here are the first few lines:

I run up and down the corridor

Ticking tasks off my list

Floor to floor

Is there is anything I have missed…..

As I conclude this editorial, the final one of our 10^th^ year of publication, I wonder what I too may have missed.

